# A time based objective evaluation of the erosive effects of various beverages on enamel and cementum of deciduous and permanent teeth

**DOI:** 10.4317/jced.55910

**Published:** 2020-01-01

**Authors:** Gayathri Rajeev, Amitha J. Lewis, Srikant N

**Affiliations:** 1 Former Student. Manipal College of Dental Sciences, Mangalore. Manipal Academy of Higher Education, Manipal; 2 Associate Professor. Department of Oral Pathology and Microbiology. Manipal College of Dental Sciences, Mangalore. Light House Hill Road, Mangalore -575001. Manipal Academy of Higher Education, Manipal; 3 Professor and Head. Department of Oral Pathology and Microbiology. Manipal College of Dental Sciences, Mangalore. Manipal Academy of Higher Education, Manipal

## Abstract

**Background:**

Erosion of the teeth is a chronic irreversible process leading to loss of surface enamel and even the dentin, in turn causing sensitivity and pain. Increased consumption of carbonated beverages remains a major cause for dental erosion. However, many of the so called safe beverages that are consumed may also have sufficiently low pH to cause dental erosion. One of the parameters to measure the dental erosion is estimation of hardness and surface roughness. Thus, this study aims to evaluate the difference in hardness and surface roughness of enamel and cementum using three beverages namely (carbonated drink, lime soda, lime juice) in deciduous and permanent teeth.

**Material and Methods:**

Ten permanent and three deciduous teeth samples each were kept in lime juice, lime soda, carbonated beverage and tap water. The VHN using Vickers hardness tester and Ra value using surface profilometer were assessed at baseline, 1 day and 10 days.

**Results:**

At the end of 10 days the decrease in hardness of enamel of permanent teeth was maximum for teeth immersed in carbonated beverage followed by lime soda and lime juice. However, in the deciduous teeth it was observed that the VHN drop was maximum at 1 day in relation to teeth immersed in carbonated beverage followed by lime juice and lime soda. The hardness of cementum decreased significantly at the end of ten days both in deciduous as well as permanent teeth.

**Conclusions:**

The present study shows that many of the most commonly used beverages like lime juice and lime soda have a sufficiently low pH to cause erosion of the enamel surface as well as that of cementum of both deciduous and permanent teeth. Though protective mechanisms do exist in the oral cavity to neutralize the acids present in these beverages, continuous usage of these beverages leads to irreversible damage to the tooth structure.

** Key words:**Dental erosion, hardness, surface roughness, permanent teeth, deciduous teeth.

## Introduction

Tooth wear is an additive, multi-factorial, lifelong process which is to a large extent irreversible. Dental erosion is a form of tooth wear which is defined as a loss of tooth substance by chemical process not involving the bacteria (1). Dental erosion according to Tencate and Imfeld (1996) “is the clinical term that is used to describe the physical results of a pathologic, chronic, localized loss of dental hard tissue that is chemically etched away from tooth surface by acid/chelation without bacterial involvement” (2). The acids responsible for tooth erosion, arise from intrinsic (such as eating disorders or gastric reflux) or extrinsic sources. One important extrinsic factor responsible for tooth erosion is high consumption of carbonated drinks and acidic foods, the frequency of which is increasing with changing life styles in the modern world (3). The acids added in various drinks help improve the palatability of the drink but at the same time contributes to erosion of tooth structure. These acids include citric acid, phosphoric acid and malic acid (4-6).

Clinically it may be difficult to diagnose erosion in the early stages. At a macroscopic level the erosion may appear as smooth, silky glazed, sometimes dull enamel with the absence of perikymata. Severe erosion may be associated with rounding off of the cusps and restorations and severe dentinal sensitivity owing to exposure of the dentinal tubules (2).

In the initial stages, erosion of the tooth leads to changes in the physical property of tooth including alterations in the surface microhardness and surface roughness. Microhardness is measured with either a Knoop or a Vickers diamond indenter. Surface roughness is vertical deviation of a real surface from its ideal form. Surface roughness was measured using a profilometer. Surface profilometry is used to measure this surface roughness which quantifies the loss of dental tissue in relation to a non-treated reference area (7).

Various studies have evaluated the erosive potential of carbonated beverages (4-6). Lime juice commonly called as “nimbu paani” in India is a frequent energy drink consumed by children and adults. Among the numerous Indian modifications of lime juice, the carbonated variant “fresh lime soda”, is considered to be a good digestive. Therefore, the aim of this study was to evaluate the erosive potential of carbonated beverage along with the commonly used beverages namely lime juice and lime soda. To the best of our knowledge this is the first time these beverages are being used for evaluation of their erosive potential.

## Material and Methods

The present study was conducted in the Department of Oral Pathology and Microbiology, MCODS, Mangalore in association with Department of Mechanical and Manufacturing engineering, M.I.T, Manipal. The study commenced only after obtaining clearance from the Institutional Ethics Committee.

**Figure 1:**
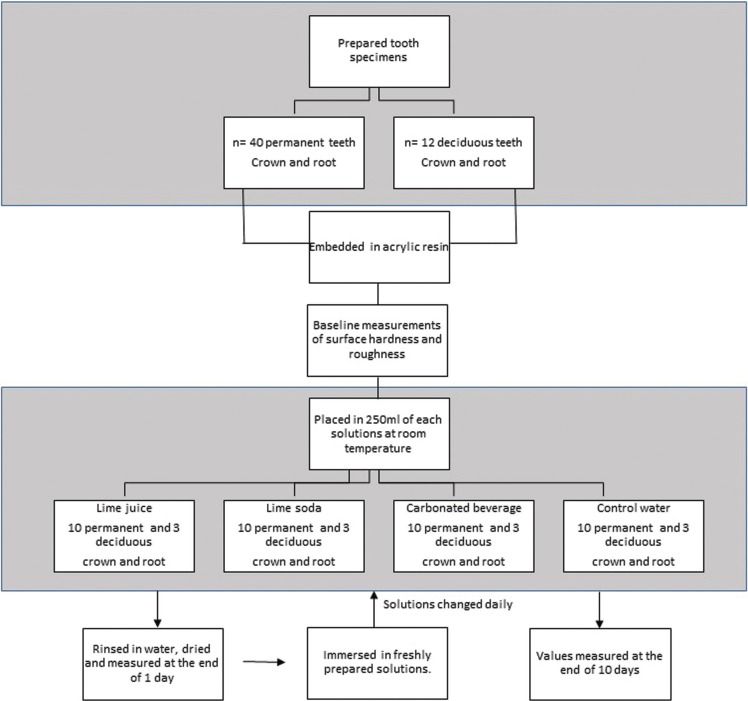
Flow diagram of the samples.

-Sample size calculation:

Based on the article by Lussi A *et al.* published in European Journal of Oral Sciences 2000, the hardness values of the aerated drinks before and after immersion in permanent teeth was 355 and 221 KHN respectively as given in Figure 1c. The mean decrease in hardness was seen to be 25.9 ±15.6 for permanent teeth.

Using the formula:

and k substituted as 6 for the comparison of 4 groups z values for beta and alpha, with a power of 80 % and an alpha error rate of 5% substituted as 0.84 and 2.63 respectively, standard deviation taken as 15.6, clinically relevant difference (d) taken as 25, we arrived at a sample of 10 per group.

-Preparation of tooth specimens:

Forty extracted permanent teeth and twelve extracted deciduous teeth were selected, carefully cleaned and stored in distilled water. Care was taken to include teeth that did not have any caries, hypocalcification or visible cracks. The teeth that did not meet the inclusion criteria were excluded from the study. As the profilometer (a Mitutoyo profilometer (Model no.SJ-301) requires a flat area for measurement, an area of 4mm2 was flattened on the buccal surface of the enamel and polished. The tooth was sectioned transversely using a slow speed diamond disc at the level of the CEJ to separate the crown and the root. Each of the crown and the root were then embedded in acrylic resin with the buccal surface faced upwards to obtain a firm base in order to facilitate the measurement of the hardness test. Each specimen was assigned a number.

-Preparation of the beverages:

The study included three beverages namely commercially available carbonated beverage, lime juice and lime soda. Tap water was used as a control. Lime juice and lime soda were prepared using juice of one lime in 250ml of water and soda water respectively. The pH of each of these beverages was measured with a pH meter (Elico LI 615 pH meter) connected to an electrode calibrated with standard solutions of pH 4.0 and 9.0, respectively.

-Baseline measurement:

Baseline microhardness measurements were performed using a Vickers indentor, Matzusawa microhardness tester (Model –MMT X 7A). Three indentations per test were performed on each specimen. The indentation load was 100g with 15s dwell time. Baseline surface roughness was measured using a Mitutoyo profilometer (Model no.SJ-301). The area used for measuring surface roughness was 1.25 mm. The roughness parameter of consideration being Ra (defined as average distance from the profile to the mean line over the length of assessment).

After baseline microhardness and surface roughness were recorded, ten permanent and three deciduous teeth samples (crown and root) were placed each in 250ml of carbonated beverage, lime soda, lime juice and water at room temperature. Water was used as a control. At the end of one day, the specimens were rinsed in water, dried and subjected to micro hardness and surface roughness tests. The values thus were recorded following which the tooth samples were immersed in the same freshly prepared solutions. The beverages were changed every day till the tenth day following which the same tests were repeated. The hardness and surface roughness were recorded after placement of the samples in their independent solutions for 0, 1 and 10 days (Fig. 1).

-Statistics:

Shapiro Wilk test for normality was performed for the parameters of surface roughness and hardness and it was found to be not statistically significant indicating a normally distributed data. Thus, one-way ANOVA and posthoc Tukey test (parametric tests) were used to assess the surface hardness and roughness of each of the beverages.

## Results

**Figure 2:**
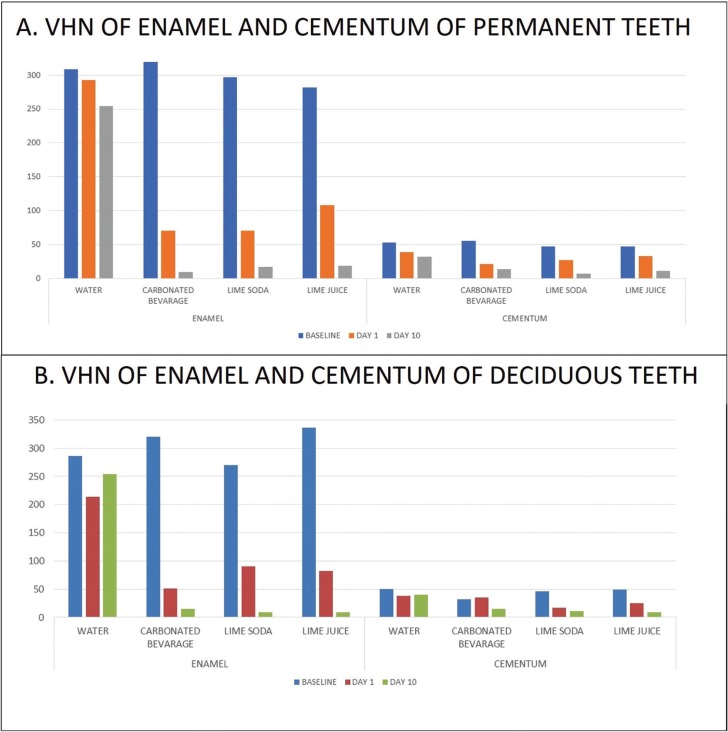
The change in the micro-hardness of enamel and cementum of permanent and deciduous teeth following immersion in different beverages at different time intervals.

The carbonated beverage had the lowest pH of 2.6, while lime juice and lime soda had values of 3.0 and 3.4 respectively and the pH of water was 6.3. The mean baseline of Vickers’s hardness numbers were 301.66 ± 44.43 for permanent and 303.01±47.90 for primary teeth. Enamel hardness decreased significantly (*p*<0.001) after immersion in all three test solutions namely carbonated beverage, lime juice and lime soda. The greatest decrease in VHN of enamel after one day was found with carbonated beverage for both permanent and primary teeth. Overall at the end of 10 days the decrease in microhardness of enamel of permanent teeth was maximum for teeth immersed in carbonated beverage (difference of 309.8 from baseline value) followed by lime soda (difference of 280.2 from baseline value) and lime juice (difference of 263 from baseline value). However, in the deciduous teeth it was observed that the VHN drop was maximum at 1 day in relation to teeth immersed in carbonated beverage (difference of 269 from baseline value) followed by lime juice (difference of 253.4) from baseline value and lime soda difference of 179.7 from baseline value) ( Table 1, Fig. 2).

**Table 1:**
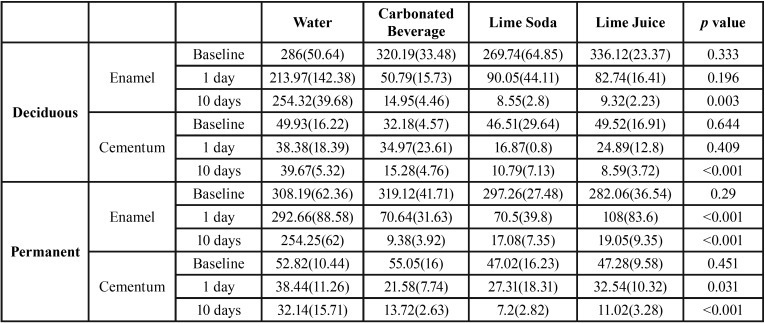
One way-anova for hardness in each immersion fluid.

The microhardness of cementum ranged between 50± 13 for permanent teeth and 44.5± 17.9 for deciduous teeth. The hardness of cementum decreased significantly at the end of ten days both in deciduous as well as permanent teeth. The decrease was seen to be maximum in lime juice (drop of 41.0 units from baseline value) followed by lime soda (drop of 35.8 units from baseline value) and carbonated beverage (16.9 VHN) in deciduous teeth and this was found to be statistically significant (*p*<0.001). At the end of day 1, the hardness of cementum of permanent teeth reduced drastically in teeth immersed in carbonated beverage. (drop of 33.5 units) ( Table 1, Fig. 2).

**Figure 3:**
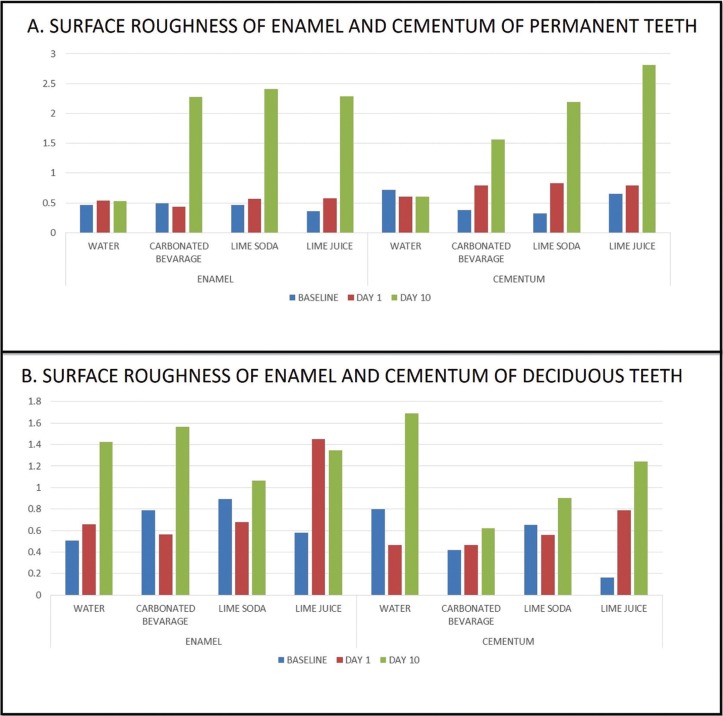
The surface roughness of enamel and cementum of permanent and deciduous teeth following immersion in different beverages at different time intervals.

The surface roughness of the enamel of permanent teeth increased significantly at the end of ten days in relation to all the test solutions. In the enamel of permanent teeth though the change in the surface roughness was not significant at day 1, there was a sudden increase in roughness at the end of 10 days. The roughness of the cementum at the end of ten days was found to be maximum in roots immersed in lime juice (2.81) followed by lime soda (2.19) and carbonated beverage (1.55) ( Table 2, Fig. 3A).

**Table 2:**
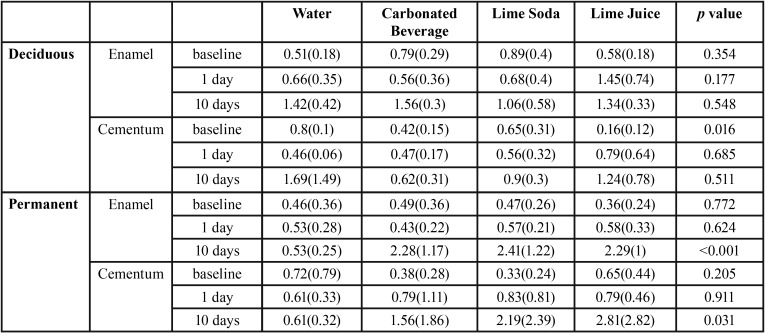
One way-ANOVA for surface roughness in each immersion fluid.

The surface roughness of the enamel and cementum of deciduous teeth increased at the end of ten days in relation to all test solutions ( Table 2, Fig. 3B). However, the results were not statistically significant (*p*>0.001)

On comparison of the enamel and cemental surface clinically at the end of ten days we found that the enamel showed a softened opaque surface, whereas such an appearance was lacking in the cementum.

Comparison of the rate of erosion in primary and permanent teeth showed insignificant results. However, primary teeth showed marginally faster rate of erosion in enamel, whereas permanent teeth showed greater loss of cementum over time.

## Discussion

Host factors are integral in regulating pH and therefore play a role in maintenance of tooth structure integrity. The hydrogen ions play an important role in demineralization by regulating the saturation levels of the minerals. Lower pH tends to promote under-saturation thereby hastening demineralization. The pH at which a solution is saturated with a particular mineral like enamel is called critical pH. Dissolution of enamel occurs when the pH falls below the pH of 5.5. (3,4,8) This study tested the pH of all the test solutions and found them to be below the critical pH. The carbonated beverage had the lowest pH of 2.6, while lime juice and lime soda had a pH of 3.02 and 3.05 respectively. The etching effects of these acidic beverages, start when they contact the enamel surface for a short period of time.

Hardness of the enamel structure can be regarded as a surrogate marker for mineral content (7). In the present study, Vickers hardness test under a load of 100g was used to assess the hardness of the enamel. Demineralization leads to surface irregularity which results in surface roughness which can be aptly measured using surface profilometry (9). Though earlier literature documents some disadvantages of stylus profilometer like inability to detect valleys narrower than the stylus tip and the risk of the diamond tip causing damage to the specimens it still can be used as a marker for roughness (10). The present study utilized both these methods to evaluate the erosive potential of the three beverages.

The greatest decrease in VHN of enamel after one day was found with carbonated beverage for both permanent and primary teeth. These results are similar to the earlier studies which shows that the carbonated drinks, which have a lower pH, correlated with higher erosive potential on immediate exposure as compared to fruit based drinks (which contain citric acid) (4,11). In a similar study conducted by Seow *et al.* they found that the hardness reduced by about 50% for lime juice and 24% in case of coca-cola (4). However, they used lime juice concentrate as compared to the diluted lime juice in our study. The variation in the erosive potential could be attributed to differences in the acid content, acid type and possible duration of contact. The carbonated beverages contain phosphoric acid in addition to the citric and carbonic acids, whereas lime juice mainly contains citric acid and lime soda contains both. Phosphoric acid, is more potent compared to the other two acids and may cause a superficial etched zone that might be lost from the tooth surface, whereas citric acid may act as a chelator capable of binding calcium from enamel or dentin, creating a higher degree of undersaturation, thus favouring demineralization. The presence of phosphoric acid in the carbonated drinks, thus may be the factor, explaining the higher erosive action of the carbonated beverage as compared to lime juice and lime soda (11,12).

Erosion may be characterized by initial softening of the enamel surface which is dependent on the time of immersion, the pH and the type of beverage/acid. Other than these, the titrable acid content, calcium chelating properties, stimulation of salivary flow are few of the factors that modify the erosive potential of these beverages (1,3). Human enamel is a highly mineralized structure. It mostly has a prismatic structure (rods and interrods) however the outermost layer is aprismatic and is to a certain extent resistant to erosion. This mineralized tissue is densely packed with hydroxyapatite crystals, the other components being water and organic material. The erosive demineralization of enamel is a centripetal process which starts with a partial loss of surface mineral/hydroxyapatite crystals. This causes increased surface roughness. If the acidic insult still continues the rod sheath is lost in turn leading to bulk mineral loss. The surface of the enamel thus shows an etched pattern. Softened layer may be present on the surface. This partial loss of mineral at the surface results in loss of hardness (softening) as was seen in our study (3,13).

The surface roughness of both the enamel and cementum also increased with time following exposure to the test solutions. The increase in surface roughness of enamel was in accordance with the study conducted by Machado *et al.* (14). On clinical examination we found that at the end of ten days the enamel showed a softened opaque surface similar to the study reported by Fujji *et al.* wherein the enamel surfaces placed in coca-cola and orange juice were visibly roughened and had lost their lustre (9). Such an appearance was lacking in the cementum. This is because in enamel there is loss of volume due to its high mineral content and less of organic component, whereas in erosion of the cementum, even when the mineral component is partially or fully dissolved the collagen structural proteins remain. So as long as the organic component is not damaged the appearance is maintained. However, during this time, the root may be susceptible to damage due to faulty tooth brushing (15,16).

Various investigators have studied the possible differences between the susceptibility of primary and permanent enamel to erosion. However, the results have been contradictory. Lussi *et al.* in his study showed that the primary teeth were initially as resistant to acids as permanent teeth (1). In the present study, the rate of erosion was faster in deciduous teeth in comparison to the permanent teeth similar to previous observations by Wang *et al*. and Haghgou *et al.* (8,17). The mineral content in primary enamel is 81.3-94.2wt% whereas for permanent enamel it is around 97% the rest of it being water and organic matrix (8).

The primary teeth may be more prone to erosion due to their disordered crystal structure and difference in porosity. The salivary flow rate in the younger children may be lower further adding to the increase in erosion (18).

One of the limitations of the study was that in order to measure the indentations during Vickers hardness testing, the surface of the enamel was flattened which lead to the loss of the outer prismatic enamel which is more resistant to corrosive effects. This also partially explains the increased rates of erosion. Secondly, it measures erosion based on enamel roughness and hardness and not on the quantity of mineral lost.

-Clinical implication 

In the modern society dietary awareness is an important issue. The consumption of carbonated beverages is quite common in the present day. The ill effects of these have been highlighted by many other studies as well (3,4,5,12). However, the fact that commonly used beverages like lime juice and lime soda that is savored by children and adults alike, in India can also cause similar changes in tooth has not received adequate attention. These beverages can not only cause erosion of enamel, but in the long run can lead to dentinal sensitivity or in severe cases, pulp exposure or even tooth fracture. As we cannot completely overlook the health benefits of citrus fruits and avoid them, we should be cognizant of their ill effects and prevent possible damage. Erosive effects of these beverages differ in individuals based on the contact area, time and flow speed due to variation in drinking habits (19). In the oral cavity the tooth is covered by a pellicle acquired from the salivary proteins. This pellicle offers some amount of resistance to erosion. In the presence of acids, this protective protein covering is washed off after a certain amount of time, exposing the crystals, due to which their protective effect ceases. There should be sufficient time for the renewal of this covering, to further withstand an acidic challenge. It is this time which is a critical factor in individuals sipping these beverages throughout the day which may explain their high erosive potential (3,11). Practices that increase the acid-tooth contact time such as ‘holding’ or ‘swishing’ the beverage in the mouth could increase the chance of erosion, and should be avoided (15,19).

When a person is swishing the drink in the mouth there is an increased agitation leading to enhanced dissolution as the semistatic layer of solution close to the enamel will be constantly replaced without reaching the saturation level (3). On comparison of the potential erosive habits in individual with high and low indices for erosion Johansson *et al.* reported that in men higher erosion correlated with higher consumption of such beverages and holding time, nearly 70% longer than individuals without erosion (20). Studies have reported that only limited mineral precipitation can occur from extraoral exposure to saliva, thus even when the teeth with softened enamel were placed in oral environment there was no significant increase in microhardness. Enamel thus is vulnerable during this time to abrasive forces such as tooth brushing. Thus brushing teeth immediately after consumption should be avoided (3,21). It has been suggested that usage of straw for drinking these beverages could be beneficial. However, it depends on the appropriate position of the straw. The straw should be directed towards the oral cavity. If it is directed towards any particular tooth surface, then an increase in erosion can occur. Temperature of the beverages could also play a role in erosion. Chilled drinks are said to be less harmful to enamel than drinks consumed at room temperature or hot (19). This is because at higher temperatures an increased solubility and diffusion coefficient rate of ions (calcium and phosphate) in aqueous solution especially with citric acids has been reported.

The usage of topical fluoride varnishes and chewing gums to increase salivary flow may have some beneficial effects in reducing the dental erosion. Topical fluoride varnishes form a protective layer on the surface of the tooth thus reducing the contact of acid with enamel and also helping in enamel demineralization (3,23).

## Conclusions

The present study shows that many of the most commonly used beverages like lime juice and lime soda have a sufficiently low pH to cause erosion of the enamel surface as well as that of cementum of both deciduous and permanent teeth. Though protective mechanisms do exist in the oral cavity to neutralize the acids present in these beverages, continuous usage of these beverages leads to irreversible damage to the tooth structure.
